# The Proper Use of Cholagogues

**Published:** 1874-03

**Authors:** W. C. Smydth

**Affiliations:** Worthington, Indiana


### THE PROPER USE OF CHOLAGOGUES.

BY DR W. C. SMYDTH, WORTHINGTON, INDIANA.

In the treatment of most of the diseases of this climate, if medicines were addressed more to arouse the secretions of the liver than is generally done, it is my opinion that many more lives would be saved, and that those that do recover would do so much more speedily than is generally the case.

When using alteratives to act upon the liver in cases of fever, or any disease of the system whatever, if the discharges from the primæ viæ are copious and of a watery consistence, it matters not if there is a secretion of bile, it is best not to let the evacuations run too far, so as to produce too much debility, but to check the bowels at once and continue mild alteratives every three or four hours, according to the nature of the disease and the condition of the patient, and have it so guarded by opium or some astringent that it may not run off by the bowels till such time as the secretions are fully aroused, and then give some mild cathartic to carry away the offending matter which may have been accumulating in that organ and the portal circulation for an indefinite time.

It will be asked, what kind of agents are best to act on and arouse the secretions of the liver? As far as my experience goes, mercury in its various forms surpasses all other means for effecting this purpose; in some cases calomel is preferable, in others blue mass, and in others chalk and mercury. It is altogether owing to the disease, and the condition of the liver accompanying it, the age, temperament and constitution of the patient.

In cases where there is high fever, with pain in the head, back and limbs, with the bowels constipated, I commence with a cathartic of calomel and rhubarb, to open the bowels freely. If this is not sufficient, I give small doses of calomel with diaphoretic powders, or opium, to produce diaphoresis, ease pains and prevent its cathartic effect till it may have time to act on the secretions. In cases of dysentery, after having opened the bowels by a mild cathartic, I use chalk and mercury, powdered opium and acetate of lead, repeated at intervals of three or four hours till the secretions are in a normal condition, and generally find my cases improve under the treatment. In diarrhœas of children and infants I pursue the same treatment, with doses suited to the ages of the patients, and generally with success, such diet always being given as will not produce irritation of the bowels, but will support the strength and nourish the system.

In typho-malarial fevers, where the bowels are in a loose condition and the liver inactive, the fever of a low grade and the tongue cracked and dry, I am now mostly in the habit of using chalk and mercury, diaphoretic powder and sulph. quinine, combined with my other remedies, to act as an alterative, diaphoretic and tonic.

Mercurials have been much abused and underrated for the past few years, from the fact of their indiscriminate use by those unacquainted with their therapeutic agency.

Salivation and sore mouths used to be so common, from the use of mercurials, that several schools of medicine sprang up in opposition to their use entirely, thereby excluding from the Materia Medica one of the best remedies in the cure of disease that belonged to it. So great was the opposition to their use, that many persons became so prejudiced against them as to refuse to administer them in any form, for fear of their constitutional effects.

This, in one respect, has had a very salutary influence in the use of the remedy, to cause a greater amount of care to be used in its administration; but now, within the last few years, since chlorate of potash has come into general use, we have the complete control of it. I have used mercury freely but cautiously ever since I entered the profession, and I have not seen a sore mouth for a dozen years, to amount to anything, for I use chlorate of potassa when necessary; and I have no hesitation in saying to my professional brethren that mercury in its various forms is a better remedy to act on the secretions of the liver and restore a normal action to that organ, with its healthful influence upon all the other organs of the system, than any other remedy in the Materia Medica. —*Med. & Surg. Rep.*

